# Evidence for an Interaction Between *NEDD4* and Childhood Trauma on Clinical Characters of Schizophrenia With Family History of Psychosis

**DOI:** 10.3389/fpsyt.2021.608231

**Published:** 2021-04-08

**Authors:** Xiao-Jiao Bi, Lei Hu, Dong-Dong Qiao, Chao Han, Meng-Meng Sun, Kai-Yan Cui, Li-Na Wang, Li-Min Yang, Lan-Fen Liu, Zhe-Yu Chen

**Affiliations:** ^1^Department of Psychiatry, Shandong Mental Health Center, Cheeloo College of Medicine, Shandong University, Jinan, China; ^2^Shandong Key Laboratory of Mental Disorders, Department of Anatomy and Neurobiology, School of Basic Medical Sciences, Shandong University, Jinan, China; ^3^Institution of Traditional Chinese Medicine Innovation Research, Shandong University of Traditional Chinese Medicine, Jinan, China

**Keywords:** childhood trauma, gene-environment interaction, *NEDD4*, phenotype, schizophrenia

## Abstract

**Background:** Neural precursor cell-expressed developmentally downregulated 4 (*NEDD4*) polymorphisms and childhood trauma (CT) are associated with schizophrenia. However, whether *NEDD4* interacts with CT on symptoms of schizophrenia remains unknown. This study aimed to investigate the gene–environment interaction effect.

**Methods:** We recruited 289 schizophrenia patients and 487 controls and genotyped rs2303579, rs3088077, rs7162435, rs11550869, and rs62043855 in their *NEDD4* gene.

**Results:** We found significant differences in the rs2303579 and rs3088077 between the two groups. Patients with the rs2303579 CC genotype had higher scores compared with other genotype (*P* = 0.026) in the test of positive schizophrenia syndrome scores, whereas patients with the rs3088077 TT (*P* = 0.037) and rs7162435 CC genotypes (*P* = 0.009) had higher scores compared with the other genotypes in the test of excitement factor. Patients with a family history of psychosis (FH+) reported higher negative scores (*P* = 0.012) than those without. Patients exposed to physical abuse (PA) reported a lower language learning and memory score (*P* = 0.017) and working memory score (*P* = 0.047) than those not. Patients exposed to sexual abuse (SA) reported a lower reasoning and problem-solving skills score (*P* = 0.025); those exposed to emotional neglect (EN) reported a lower social cognition score (*P* = 0.044); and those exposed to physical neglect reported a lower social cognition score (*P* = 0.036) but higher visual learning and memory score (*P* = 0.032). Rs3088077 could interact with EN to increase risk for schizophrenia. Optimal model rs62043855 × EA, rs3088077 × rs7162435 × rs11550869 × SA × EN and rs2303579 × rs7162435 × rs11550869 × rs62043855 × EA × PA could explain positive symptom, excitement symptom and working memory, respectively, in FH+ group.

**Conclusion:** The study highlighted that the combined interaction of *NEDD4* and CT may be associated with symptoms of schizophrenia especially for those with FH+.

## Introduction

Schizophrenia has a lifetime risk of ~1% and is manifested by a disruption in cognition and emotion, along with negative and positive symptoms. Genetic factors account for more than 80% of the variance in susceptibility, and risk likely results from multiple loci with small effects (1). According to the neurodevelopment hypothesis, variations in the genes implicated in neuronal function and synaptic plasticity mechanisms may be associated with schizophrenia (2, 3). Studies have confirmed that the interaction of genes and environmental factors can affect the symptoms of psychological diseases, whereas childhood abuse is an important environmental factor that can affect the occurrence and development of schizophrenia (4). Systematic reviews of the literature and meta-analyses have demonstrated a clear relation between childhood trauma (CT) and the severity of psychotic symptom, increased prevalence of substance use, and a more refractory and prolonged illness course (5). Meanwhile the impact of CT on symptoms of schizophrenia may be enhanced due to genetic effects, particularly in the negative sub-domai (6), with a family history of psychosis (FH+) being considered the strongest genetic risk factor for schizophrenia. The course and severity of schizophrenia and long-term occupational outcomes are reportedly affected by FH+ (7). For FH+ and exposure to pyelonephritis increases by five times the risk of schizophrenia (8).

The neural precursor cell expressed developmentally down-regulated 4 (NEDD4) is a ubiquitin ligase that is critical to all stages of neuronal development, such as neuronal cell fate determination, neurite outgrowth, axon guidance, and neuronal cell survival in the developing mammalian brain (9–12). The NEDD4 protein involved in the pathological process of neurodegeneration diseases, such as Parkinson's, has been found both in animal models (13) and postmortem studies (14). Srinivasan et al. found that rs1912403 in *NEDD4* is a risk factor for Parkinson's disease in white people (15), Nonetheless, whether *NEDD4* plays a beneficial or detrimental role remains controversial.

Studies associating *NEDD4* and mental illness are relatively lacking. Lien et al. using genomic linkage, suggested that 15q21.3, where *NEDD4* is located, is related to the social isolation and introverted score of non-schizophrenic relatives (16). Jahanshad et al. found that rs17819300 and rs1781928 of *NEDD4* are related to abnormal brain connections (17). Furthermore, Warnica et al. in a study on the micro-RNA of copy number variation in patients with schizophrenia, reported that the *NEDD4* gene may be one of the target genes of schizophrenia (18). Our previous study revealed that the rs2303579 and rs62043855 loci in *NEDD4* are associated with cognitive dysfunction in schizophrenia patients in the Chinese Han population (19).

As gene–environment interactions (G × Es) are increasingly assumed to play a crucial role in schizophrenia (20) studies have investigated the role of multiple gene variants and their combined effects on susceptibility and phenotype of schizophrenia. To our knowledge, however, whether the interaction between *NEDD4* and childhood abuse affects the clinical symptoms of schizophrenia patients remains unknown. Thus, the current study aimed to investigate the potential involvement of G × Es of *NEDD4* and CT on the clinical symptoms of schizophrenia.

## Materials and Methods

### Sample

The study sample included 289 patients with schizophrenia recruited from Shandong Mental Health Center and 487 healthy controls (HCs) recruited from the community via advertisements. All Chinese Han patients aged 18–65 years fulfilling the International Statistical Classification of Diseases and Related Health Problems 10th Revision (ICD-10) criteria for schizophrenia were recruited. They had a certain level of education to understand and comply with the relevant requirements of the study, not taken any antipsychotics for at least 1 month before being recruited, and not undergone convulsive electroconvulsive therapy within 6 months. The diagnosis, along with a review of psychiatric case records, was independently checked and verified by two senior psychiatrists. Patients with serious physical diseases or those who fulfilled ICD-10 criteria for organic mental disorders, mental and behavioral disorders from psychoactive substances, schizoaffective disorder, or affective disorder were excluded. Women who were pregnant, lactating, or planning to become pregnant, and those who participated in other clinical research within 4 weeks before enrolment were also excluded.

### Clinical Assessment

#### Positive and Negative Syndrome Scale (PANSS)

We used PANSS to obtain the clinical characteristics of schizophrenia. After the patients' admission to hospital, psychiatrists who passed the consensus assessment performed the rating scale within 3 days. To conduct further factor analyses, we used the five components of PANSS, which are positive, negative, excitement, anxiety/depression, and cognitive defect (21).

#### Childhood Trauma Questionnaire-Short Form (CTQ-SF)

We employed a short-form version of CTQ-SF to retrospectively elicit information on a range of maltreatment experiences from schizophrenia participants. The CTQ-SF is a 28-item tool using a five-point Likert scale ranging from 1 (*never true*) to 5 (*very often true*). The reliability and validity of the Chinese version of CTQ-SF have been analyzed by Xing-Fu et al. (22) and Zhang (23) and their results suggested that it is a good psychometric instrument for the evaluation of Chinese childhood abuse. Five types of CT are measured in the CTQ-SF: emotional abuse (EA), physical abuse (PA), sexual abuse (SA), emotional neglect (EN), and physical neglect (PN). In the current study, the CTQ-SF cut-off scores were as follows: PA ≥ 8, SA ≥ 6, EA ≥ 9, PN ≥ 8, and EN ≥ 10 (24). We used low-to-moderate exposure cut-off scores to capture cases with even the lowest severity of CT. We handed out the questionnaire the day after the patient was admitted and asked that it be returned within 3 days.

#### Matrics Consensus Cognitive Battery (MCCB)

We used the Chinese version of MCCB to evaluate the patients' cognitive function. This tool consists of 10 sub-tests covering seven dimensions, namely, assessment of processing speed, attention and alertness, working memory, language learning and memory, visual learning and memory, reasoning and problem-solving skills, and social cognition (25). According to the patient's condition, we collected MCCB data within 7 days of enrolment to the study.

### Genotyping

All of the DNA samples we used originated from venous blood (5 mL) in EDTA-containing tubes. The improved potassium iodide method was used to extract genomic DNA after processing the blood samples. Five single nucleotide polymorphisms (SNPs) of *NEDD4*, namely, rs2303579, rs3088077, rs7162435, rs11550869, and rs62043855, were genotyped using TaqMan probe system reported in our previous study (26).

### Statistics

Data were analyzed using IBM SPSS Statistics for Windows, Version 20.0 and generalized multifactor dimensionality reduction (GMDR Software Beta, version 0.7). We used chi-squared tests to analyze the Hardy–Weinberg equilibrium (HWE) and differences between the various qualitative data of the study subjects, including sex, FH, course of disease, genotype, and CTQ score. Meanwhile, differences in the PANSS and MCCB scores of the patients in the case group were compared using one-way analysis of variance. To control for the impact of sex, age, and course of disease on clinical symptoms, we used clinical symptoms as dependent variables and the five SNP genotypes as independent variable in conducting analysis of covariance (ANCOVA). These continuous variables were presented as mean ± standard deviation.

The interaction between the *NEDD4* gene and childhood abuse was first analyzed by logistic regression. Heterozygous genotype was set as reference category, equal to 0, to correspond to the other two dummy variables in the five SNPs. The dummy genotypes variables were used as the dependent variables, whereas environmental factors were used as the independent variables in the logistic regression analysis. Second, the G × Es on the clinical symptoms of schizophrenia patients grouped by FH and course of disease were analyzed by GMDR, a free resource accessible at http://www.healthsystem.virginia.edu/internet/Addiction-Genomics/ (27). A Age and sex were included as covariates in all the interactions analyses. Given that the selected traits used for score calculation were quantitative ones, we selected the linear regression method to calculate the statistical scores. All interactions were tested using 10-fold cross-validations in an exhaustive search that considered all possible combinations. The model with the highest testing balance accuracy and cross-validation consistency was selected as the optimal model. *P* < 0.05 was considered as statistically significant.

## Results

### Demographics, Sample Characteristics

Among these 289 patients with schizophrenia, there were 185 females with a mean age of 35.21 ± 11.464 years and 104 males with a mean age of 29.21 ± 9.031 years. 487 HCs (female = 282) aged 32.53 ± 10.386 were enrolled in the study. There were no statistically significant differences found between patients and HCs for sex and age (*P* > 0.05). Of those patients with schizophrenia, there were 200 patients with FH+ and another 89 cases without family history of psychosis (FH–). Besides, when grouped by course of schizophrenia, we found 70 patients were first episode and 219 were recurrent. All of those patients completed MCCB and PANSS and all the blood samples were successfully genotyped. We did not find any significant differences between genotype distributions and sex, FH, course of disease, respectively (Table 1).

**Table 1:** One-way analysis of among genotype distributions and gender, FH, course of disease.

**SNP**	**Genotype**	**Sex**		**χ^2^**	***P***	**FH**		**χ^2^**	***P***	**Course of disease**		**χ^2^**	***P***
		**Male (104)**	**Female (185)**			**+(200)**	**–(89)**			**Fist (70)**	**Recurrent (219)**		
rs2303579	CC (91)	33	58	0.298	0.862	62	29	0.452	0.798	24	67	0.835	0.659
	TT (69)	23	46			50	19			14	55		
	CT (129)	48	81			88	41			32	97		
rs3088077	CC (106)	42	64	2.359	0.307	74	32	0.424	0.809	29	77	1.492	0.474
	TT (49)	20	29			32	17			9	40		
	CT (134)	42	92			94	40			32	102		
rs7162435	TT (144)	49	95	0.652	0.722	102	42	0.707	0.702	29	115	2.619	0.270
	CC (24)	10	14			15	9			7	17		
	TC (121)	45	76			83	38			34	87		
rs11550869	GG (12)	3	9	1.661	0.436	11	1	3.117	0.21	3	9	0.009	0.996
	CC (210)	80	130			142	68			51	159		
	GC (67)	21	46			47	20			16	51		
rs62043855	TT (123)	47	76	1.194	0.551	85	38	0.453	0.797	34	89	2.649	0.266
	GG (40)	16	24			26	14			6	34		
	TG (126)	41	85			89	37			30	96		

*FH, family history*.

A total of 289 CTQ-SF questionnaires were distributed out of which 136 completed questionnaires were returned and used for the study. Among them, 36 were males, 100 were females, 86 were FH positive, 50 were negative, 42 were first-episode patients, and 94 were relapsed patients. Those who exposed to five types of CT based on our cut-off scores did not differ significantly from course of disease, FH (Supplementary Table 1). However, significant differences were found between childhood emotional neglect and sex with female patients reporting more emotional neglect (χ^2^ = 13.926, *P* = 0.000368) (Table 2).

**Table 2:** Analysis of CTQ and sex.

**CTQ**		**Sex**		**χ^2^**	***P***
		**Male (36)**	**Female (100)**		
EA	None (69)	23	46	3.389	0.081
	Exposed (67)	13	54		
PA	None (99)	26	73	0.008	1
	Exposed (37)	10	27		
SA	None (81)	25	56	1.986	0.172
	Exposed (55)	11	44		
EN	None (36)	18	18	13.926	0.000368^*^
	Exposed (100)	18	82		
PN	None (7)	2	5	0.017	1
	Exposed (129)	34	95		

* *P < 0.05*.

*CTQ, Childhood Trauma Questionnaire; EA, emotional abuse; PA, physical abuse; SA, sexual abuse; EN, emotional neglect; PN, physical neglect. The bold values means a form of highlighting P < 0.05*.

### Distribution of 5 SNPs in *NEDD4* in Patients With Schizophrenia and HCs

All SNPs in testing HWE were not statistically significant (data not show). The genotype frequencies of rs2303579 (χ^2^ = 11.769, *P* = 0.003) and rs3088077 (χ^2^ = 18.382, *P* = 0.000102) loci were significantly different between the patients group and HCs, while the genotype frequencies of rs7162435 (χ^2^ = 5.289, *P* = 0.071), rs11550869 (χ^2^ =1.041, *P* = 0.594), and rs62043855 (χ^2^= 1.473, *P* = 0.479) loci were not significantly different (Table 3).

**Table 3:** Distribution of 5 SNPs in *NEDD4* in patients with schizophrenia and controls.

**SNP**	**Genotype**	**HCs (487)**	**Patients (289)**	**χ^2^**	***P***
rs2303579	CC	191	91	11.769	0.003^*^
	TT	71	69		
	CT	225	129		
rs3088077	CC	252	106	18.382	0.0001^*^
	TT	50	49		
	CT	185	134		
rs7162435	TT	283	144	5.289	0.071
	CC	38	24		
	TC	166	121		
rs11550869	GG	14	12	1.041	0.594
	CC	364	210		
	GC	109	67		
rs62043855	TT	224	123	1.473	0.479
	GG	55	40		
	TG	208	126		

* *P < 0.05*.

*NEDD4, Neural precursor cell expressed developmentally down-regulated 4. The bold values means a form of highlighting P < 0.05*.

### *NEDD4* Genotype Distributions, CTQ, FH, and Symptoms of Schizophrenia

One-way analysis of variance was used to compare the scores of PANSS, MCCB among the patients with three different genotypes of five SNPs locus in *NEDD4* gene, with or without five types of CTQ, FH+ or FH-. We found that patients with rs2303579 CC genotype had significantly higher scores (33.03 ± 4.841) than other genotypes (F = 3.688, *P* = 0.026) in the test of positive scores. Rs3088077 TT genotype (15.31 ± 3.825, F = 3.33, *P* = 0.037) and rs7162435 CC genotype (15.58 ± 6.043, F = 4.783, *P* = 0.009) both had significantly higher scores than other genotypes in the test of excitement scores, while no significant difference was found in rs11550869 and rs62043855 (Table 4). Additionally, FH+ patients got higher negative scores (32.91 ± 7.769, F = 6.366, *P* = 0.012) than those FH- patients (Table 4). While we did not find any significant differences between FH+ and FH- groups in MCCB scores (Supplementary Table 2). The ANCOVA results were consistent with the results of the one-way analysis of variance. After controlling for the effects of sex, age, and disease course, the genotype of rs2303579, rs3088077, and rs7162435 were related to scores of positive and excitement symptoms, respectively (Supplementary Table 3).

**Table 4:** One-way analysis of variance of PANSS scores in terms of genotype and FH.

**SNP**	**Genotype**	**Positive**	**Negative**	**Excitement**	**Anxiety depression**	**Cognitive defect**
rs2303579	CC (91)	33.03 ± 4.841	31.85 ± 8.166	13.96 ± 4.000	12.26 ± 3.197	8.93 ± 2.289
	TT (69)	31.45 ± 4.610	33.29 ± 7.098	14.72 ± 3.646	12.81 ± 2.987	9.59 ± 1.842
	CT (129)	31.33 ± 4.975	31.81 ± 7.040	13.76 ± 4.202	12.05 ± 3.024	9.15 ± 1.957
	F	3.688	1.019	1.338	1.377	2.095
	*P*	0.026^*^	0.362	0.264	0.254	0.125
rs3088077	CC (106)	32.19 ± 4.926	32.59 ± 7.689	14.06 ± 4.260	12.07 ± 3.255	9.1 ± 2.221
	TT (49)	32.02 ± 4.948	31.49 ± 8.109	15.31 ± 3.825	12.16 ± 2.726	9.49 ± 1.839
	CT (134)	31.61 ± 4.868	32.1 ± 6.979	13.59 ± 3.812	12.54 ± 3.053	9.14 ± 1.985
	F	0.429	0.383	3.33	0.753	0.653
	*P*	0.651	0.682	0.037^*^	0.472	0.521
rs7162435	TT (144)	31.79 ± 4.940	32.20 ± 7.453	14.44 ± 3.741	12.15 ± 2.828	9.33 ± 2.028
	CC (24)	32.54 ± 4.170	30.50 ± 5.680	15.58 ± 6.043	12.58 ± 3.966	8.25 ± 1.894
	TC (121)	31.88 ± 4.995	32.48 ± 7.703	13.28 ± 3.713	12.43 ± 3.175	9.20 ± 2.072
	F	0.241	0.712	4.783	0.389	2.915
	*P*	0.786	0.492	0.009^*^	0.678	0.056
rs11550869	GG (12)	32.25 ± 4.693	34.92 ± 9.568	16.08 ± 3.423	12.00 ± 1.809	9.58 ± 2.353
	CC (210)	31.74 ± 4.880	32.09 ± 7.102	14.06 ± 4.097	12.20 ± 2.872	9.11 ± 2.013
	GC (67)	32.31 ± 5.013	31.97 ± 8.015	13.66 ± 3.792	12.66 ± 3.796	9.34 ± 2.122
	F	0.383	0.858	1.871	0.607	0.55
	*P*	0.682	0.425	0.156	0.546	0.578
rs62043855	TT (123)	32.36 ± 5.258	32.5 ± 7.846	14.2 ± 4.364	12.23 ± 3.306	9.08 ± 2.296
	GG (40)	31.8 ± 4.847	32.1 ± 7.313	14.53 ± 3.630	12.83 ± 3.071	9.53 ± 1.894
	TG (126)	31.47 ± 4.523	31.89 ± 7.079	13.75 ± 3.779	12.21 ± 2.841	9.18 ± 1.835
	F	1.037	0.209	0.71	0.674	0.706
	*P*	0.356	0.811	0.493	0.51	0.494
FH	+(200)	31.68 ± 5.116	32.91 ± 7.769	13.84 ± 4.257	12.36 ± 3.180	9.27 ± 2.170
	–(89)	32.38 ± 4.339	30.54 ± 6.332	14.53 ± 3.388	12.18 ± 2.839	9.00 ± 1.745
	F	1.287	6.366	1.813	0.199	1.069
	*P*	0.258	0.012^*^	0.179	0.655	0.302

* *P < 0.05*.

*PANSS, The Positive and Negative Syndrome Scale; FH+, with family history of psychosis; FH–, without family history of psychosis. The bold values means a form of highlighting P < 0.05*.

When it came to CTQ, no significant difference between patients with or without five types of CTQ was found in any test of PANSS (Supplementary Table 4). But we found that those patients who exposed PA got a significantly lower language learning and memory score (42.27 ± 10.322, F = 5.866, *P* = 0.017) and significantly lower working memory score (87.24 ± 19.484, F = 4.016, *P* = 0.047) than who not (Table 5). In addition, those patients who exposed SA got a significantly lower reasoning and problem solving skills score (41.51 ± 9.601, F = 5.129, *P* = 0.025), those exposed EN got a significantly lower social cognition score (47.17 ± 11.759, F = 4.150, *P* = 0.044), and those exposed PN got a significantly lower social cognition score (47.88 ± 11.200, F = 4.487, *P* = 0.036) but a significantly higher visual learning and memory score (44.33 ± 11.758, F = 4.671, *P* = 0.032) compared with the other groups (Table 5).

**Table 5:** One-way analysis of variance of MCCB scores in terms of CTQ.

**CTQ**		**Processing speed**	**Language learning and memory**	**Reasoning and problem solving skills**	**Visual learning and memory**	**Social cognition**	**Attention and alertness**	**Working memory**
EA	None (69)	140.87 ± 23.50	45.91 ± 11.085	44.30 ± 9.737	44.93 ± 11.487	50.06 ± 11.068	44.3 ± 9.819	95.78 ± 20.413
	Exposed (67)	137.69 ± 25.10	45.55 ± 9.685	43.22 ± 9.802	42.7 ± 12.087	46.6 ± 11.588	42.72 ± 10.370	90.07 ± 20.544
	F	0.583	0.041	0.416	1.213	3.174	0.841	2.641
	*P*	0.446	0.84	0.52	0.273	0.077	0.361	0.106
PA	None (99)	141.48 ± 24.873	47.03 ± 10.154	43.79 ± 9.456	44.58 ± 11.586	48.92 ± 11.795	44.29 ± 10.288	95.11 ± 20.693
	Exposed (37)	133.46 ± 21.798	42.27 ± 10.322	43.73 ± 10.627	41.84 ± 12.276	46.84 ± 10.340	41.46 ± 9.356	87.24 ± 19.484
	F	2.99	5.866	0.001	1.456	0.894	2.143	4.016
	*P*	0.086	0.017^*^	0.975	0.23	0.346	0.146	0.047^*^
SA	None (81)	141.48 ± 24.471	46.8 ± 10.955	45.31 ± 9.603	44.62 ± 12.194	49.47 ± 10.164	44.85 ± 8.939	94.22 ± 19.937
	Exposed (55)	136.09 ± 23.809	44.16 ± 9.351	41.51 ± 9.601	42.67 ± 11.192	46.71 ± 12.971	41.56 ± 11.380	91.13 ± 21.597
	F	1.624	2.134	5.129	0.89	1.927	3.546	0.738
	*P*	0.205	0.146	0.025^*^	0.347	0.167	0.062	0.392
EN	None (36)	143.31 ± 24.869	47.67 ± 12.224	43.78 ± 11.087	47.00 ± 10.085	51.64 ± 9.828	44.56 ± 7.817	97.44 ± 21.840
	Exposed (100)	137.86 ± 24.003	45.04 ± 9.607	43.77 ± 9.280	42.69 ± 12.198	47.17 ± 11.759	43.15 ± 10.801	91.36 ± 20.006
	F	1.337	1.703	0.000017	3.602	4.15	0.512	2.332
	*P*	0.25	0.194	0.997	0.06	0.044^*^	0.476	0.129
PN	None (7)	135.29 ± 29.876	41.00 ± 10.909	48.71 ± 7.455	34.57 ± 8.696	57.14 ± 12.734	42.29 ± 12.065	97.86 ± 13.993
	Exposed (129)	139.52 ± 24.044	45.99 ± 10.335	43.5 ± 9.808	44.33 ± 11.758	47.88 ± 11.200	43.59 ± 10.022	92.71 ± 20.907
	F	0.201	1.541	1.91	4.671	4.487	0.11	0.413
	*P*	0.655	0.217	0.169	0.032^*^	0.036^*^	0.741	0.521

* *P < 0.05. The bold values means a form of highlighting P < 0.05*.

### Interaction Between *NEDD4* Gene and Childhood Abuse on Symptoms of Schizophrenia

The G × Es between 5 SNPs on *NEDD4* gene and 5 types of childhood abuse were tested using both logistic regression and GMDR. Our logistic regression results showed that rs3088077 could interact with environment factor EN (OR = 4.626, *P* = 0.044) (Table 6) in increasing the risk rs3088077 for schizophrenia. No interaction was found between other SNPs and 5 types of childhood abuse.

**Table 6:** Interaction between rs3088077 and CTQ by Logistic regression.

**Variables**	**B**	**SE**	**Wald**	***P***	**OR**	**95% CI**
None EA	−0.027	0.570	0.002	0.963	0.974	(0.319,2.977)
None PA	−1.113	0.733	2.306	0.129	0.328	(0.078,1.382)
None SA	−0.232	0.611	0.144	0.704	0.793	(0.239,2.627)
None EN	1.532	0.759	4.069	0.044^*^	**4.626**	**(1.044,20.489)**
None PN	0.424	1.179	0.129	0.719	1.528	(0.152,15.391)

*Reference category = TT*.

* *P < 0.05. The bold values means a form of highlighting P < 0.05*.

Using the GMDR, controlling for sex and age as covariates, we analyzed the interactions between *NEDD4* and childhood abuse on symptoms of schizophrenia in five components of PANSS and seven dimensions of MCCB. We tested all the combinations within the two aspects in FH+ and FH– subgroups. As the results shown in Table 7, we found the optimal model rs62043855 × EA which had a cross-validation consistency of 10/10 (*P*^‡^ = 0.0107, *P*^§^ = 0.002) could explain positive symptoms, the optimal model rs3088077 × rs7162435 × rs11550869 × SA × EN which had a cross-validation consistency of 10/10 (*P*^‡^ = 0.001, *P*^§^ = 0.000) could explain excitement symptoms, the optimal model rs2303579 × rs7162435 × rs11550869 × rs62043855 × EA × PA which had a cross-validation consistency of 8/10 (*P*^‡^ = 0.0107, *P*^§^ = 0.000) could explain working memory in FH+ group.

**Table 7:** GMDR analyses on *NEDD4*-CTQ interactions on clinical symptoms in schizophrenia patients grouped by FH.

**Modes**	**Training accuracy**	**Testing accuracy**	**Cross-validation consistency**	***P*^‡^**	***P*^§^**
rs62043855 × EA	0.6976	0.6672	10/10	**0.0107**	**0.002**
rs3088077 × rs7162435 × rs11550869 × SA × EN	0.8799	0.7063	10/10	**0.001**	**0.000**
rs2303579 × rs7162435 × rs11550869 × rs62043855 × EA × PA	0.9001	0.7015	8/10	**0.0107**	**0.000**

‡ *P < 0.05 and based on sign test*.

§ *P < 0.05 and based on permutation test. The bold values means a form of highlighting P < 0.05*.

## Discussion

There is indirect evidence supporting the inference that the *NEDD4* gene might interact with CT for schizophrenia. However, to the best of our knowledge, the present study is the first to link the *NEDD4* gene, CT, and schizophrenia. We used two different clinical symptom assessment methods to evaluate the clinical symptoms and two different statistical methods to explore the effect of G × Es. Our results indicated that the interaction between *NEDD4* and CT may play an essential role in the development of the clinical symptoms of schizophrenia.

Consistent with the results of previous studies, our findings indicate a relation between *NEDD4* and susceptibility to schizophrenia. Among the five SNPs of *NEDD4*, the polymorphism rs3088077 of 3'UTR and exonic polymorphism rs2303579 showed significant associations with schizophrenia. This connection is possible from the functional perspective of *NEDD4*. The NEDD4 protein is a critical ubiquitin ligase to many biological functions of the nervous system (28). It regulates the turnover and trafficking of ion channels and G protein-coupled receptors present in neurons, such as the GluA1 AMPA receptor, GluN2D-containing NMDA receptor (29, 30), voltage-gated sodium channels, calcium channels, and the newly discovered metabotropic glutamate receptor 7 (31). A recent study also found that NEDD4 could regulate hydrogen peroxide-induced cell proliferation and death through the inhibition of Hippo signaling in human bone marrow-derived stem cells (32). The effects of NEDD4 on the neurotransmitters modulate long-term potentiation and long-term memory (33), leading to the impairment of prefrontal cortex-mediated cognitive functions (34, 35). Our research have showed links between symptoms of schizophrenia such as excitement and cognitive dysfunction and several SNPs in *NEDD4* (19, 26).

The abnormal expression of the *NEDD4* gene may cause abnormalities in the nervous system, which may cause clinical symptoms in patients with schizophrenia. One animal study revealed that levels of NEDD4 and mRNA are reduced in the brain of mice fed with antipsychotic drugs, suggesting that NEDD4 may be a target for new drug interventions (36). Given the current understanding of the biological function NEDD4, NEDD4 may be inferred to be related with the development of not only schizophrenia but also other mental diseases. Indeed, a recent article explored the possible relation between *NEDD4* and depression. Maternal immune activation suppressing NEDD4-related signal pathways has been shown to impede offspring's dendrite development and cause depressive-like behaviors (37). However, clinical studies on the correlation between *NEDD4* and depression have not been conducted.

Our findings not only confirmed the possible connection between *NEDD4* and schizophrenia but also explored the optimal model of *NEDD4* × childhood abuse interaction on schizophrenic clinical symptoms. Childhood abuse has always been an environmental factor of great concern (38). The present study underlined that PA, SA, EN, and PN, determined through the CTQ-SF, were related to cognitive symptoms, consistent with previous findings showing childhood adversity as a risk factor for schizophrenia (39) and EN and PN being associated with cognitive profiles (40).

Meanwhile, previous studies have found a positive dose-response relation between SA and auditory hallucinations (41) but our results failed to show a link between CTQ and positive symptoms as well as the other factors assessed by PANSS. From our perspective, it is difficult for a single adverse experience to act on a single symptom in schizophrenia, a complicated disease. The correlation between environmental factors and symptoms calls for an evaluation of the risk factors, provision of preventative interventions, and creation of a better environment among patients with schizophrenia (42). Given that biological factors cannot compensate for all the adverse environmental factors, when the biological aspect cannot be handled, the disease will appear. We emphasized G × Es for this reason.

Interestingly, our results showed that women reported significantly more EN than men. Individuals who do not develop health-harming behaviors are more likely to have experienced safe, nurturing childhoods (43), whereas girls who have experienced EN have increased relative risk of health-related risky behaviors, such as depression, suicidal ideation, planned suicide, brawls, drunkenness, smoking, and abnormal eating behaviors (44). All of these results re-emphasize the importance of the environment. Emotional satisfaction may reduce the incidence of health-related risky behavior and mental illness, especially for women.

Regarding G × Es, a growing body of evidence suggests that early environmental risk factors and genetic predispositions for psychiatric disorders have an important influence on schizophrenia (20). Early environmental risk factors include maternal environmental risk factors (45) and CT (6). In our study, we found that *NEDD4* could enhance the effect of EN, more apparently so in FH+ patients. When FH was used as a grouping condition, the combination of rs62043855 × EA, rs3088077 × rs7162435 × rs11550869 × SA × EN and rs2303579 × rs7162435 × rs11550869 × rs62043855 × EA × PA explained positive symptoms, excitement symptoms and working memory, respectively, in FH+ group. Consistent with previous findings, we found that many genes interacted with CT to affect symptoms of schizophrenia. For example, EA interacts with the forkhead box P2 gene, involved in the development of speech and language, with respect to auditory verbal hallucinations in schizophrenia (46). We also found that the interaction between *NEDD4* and CT had a certain impact on the clinical symptoms of schizophrenia. Whether the strengthening effect of *NEDD4* on the negative environment has a role in other mental and psychological diseases, such as depression, needs to be explored further.

FH+, which acts as another important environmental risk factor with regard to schizophrenia, can be targeted in interventions. FH+ may affect patients' clinical symptoms in many ways, such as in the aspects of family growth environment and parent–child attachment. Our results showed that patients with FH+ had a higher score for negative symptoms. Regarding this finding, we have two speculations. Firstly, the genetic factors of patients with FH+ can promote social withdrawal and emotional indifference in patients with schizophrenia. Secondly, patients with FH+ may have more CT in their growing environment. As patients had not learned good ways of expressing emotions, they would tend to shrink back when sick. Therefore, our results further emphasized the importance of early intervention. FH is an obvious and reliable sign. For such families, it is necessary to provide good family and social support for the young members.

Psycho-educational programs (47), family interventions (48), and other psychosocial interventions should become essential components of the effective treatment of schizophrenia. Specifically, interventions can proceed from the following aspects. Firstly, from the patient's perspective, increased disease health education can improve treatment compliance and reduce stigma. By improving individualized treatment measures for patients through precision medicine and other methods combined with supportive psychotherapy, health professionals can improve patients' confidence in the treatment, social function, and outcomes. Secondly, from the aspect of the family, for FH+ patients, health professionals can expand disease health education and family therapy to improve the family members' understanding of the patient and the disease. The development of *NEDD4*-related locus testing items is necessary, particularly for the next generation who show positive susceptibility genotypes. More attention should be paid to the childhood abuse experience. By improving family support, providing good family atmosphere, improving parent–child interaction, and reducing bad family relations and attachment relationship affects, health professionals can help reduce the impact of FH and G × Es on the next generation. Lastly, in terms of the government and society, although China has made considerable progress in the management of severe mental illnesses, its investment in the treatment and rehabilitation of schizophrenia should be increased, along with the rehabilitation exercise measures and designated assistance for patients in the community. Alternative ways to reduce the financial and psychological burden of patients' families should likewise be explored.

Our study had several limitations. Firstly, we only chose five SNPs of *NEDD4* related to neurodevelopment based on current research, and only 136 patients with gender imbalance completed the CTQ-SF, which resulted in the reduction of our sample size. Secondly, the number of subjects required to obtain proper power for identifying G × Es in GMDR remains unknown (49). Moreover, our G × Es model was conducted in a case-only sample, which could estimate the interaction but not the main effect. Finally, the interaction model by GMDR was a statistical model; the biological effect of the combined G × Es remains unclear. These issues thereby limit the strength of our conclusions and data interpretation.

## Conclusion

Our study highlighted the importance of the combined interactions of *NEDD4* and CT in the clinical symptoms of patients with schizophrenia. We provided evidence for the need to pay more attention on patients with FH+: FH+ patients with *NEDD4* gene susceptibility who have experienced more CT may have more severe clinical symptoms. These G × Es models may better elucidate schizophrenia, which will help clinicians assess comprehensively the symptoms of patients and personalize diagnoses and therapeutic options. Meanwhile, preventing CT for children with FH of psychosis may reduce the risk of the disorder and the severity of clinical symptoms. However, given the limitations of our study, the relation between *NEDD4* and psychiatric disorders still needs to be explored further, and the impact of its interaction with CT on clinical symptoms still requires further confirmation by more clinical studies.

## Data Availability Statement

The datasets generated for this study are available on request to the corresponding author.

## Ethics Statement

The studies involving human participants were reviewed and approved by The Ethical Committee of Shandong Mental Health Center. The patients/participants provided their written informed consent to participate in this study.

## Author Contributions

L-FL supervised the whole research project. Z-YC was responsible for project design and quality control. X-JB did the literature review, the genotyping, and the statistical analyses. LH contributed to quality control and training of this subject. CH contributed to the genotyping. M-MS is responsible for the database establishment and management. D-DQ, K-YC, L-NW, and L-MY contributed to the sample collection and clinical assessment. All authors contributed to the interpretation of results and have worked on the preparation of the final version of the manuscript.

## Conflict of Interest

The authors declare that the research was conducted in the absence of any commercial or financial relationships that could be construed as a potential conflict of interest.
