# Supplementary material for: Evidence for an Interaction Between NEDD4 and Childhood Trauma on Clinical Characters of Schizophrenia With Family History of Psychosis
Source: Front Psychiatry. 2021 Apr 8;12:608231. doi: 10.3389/fpsyt.2021.608231 (PMC8060471; doi:10.3389/fpsyt.2021.608231)
Supplement: Supplementary file 1 [file Table_1.DOC]

**Supplementary Table 1. Analysis of childhood trauma and course of disease, FH**

| CTQ | FH | | F | *P* | Course of disease | | F | *P* |
| --- | --- | --- | --- | --- | --- | --- | --- | --- |
|  | +(86) | -(50) |  |  | Fist(42) | Recurrent(94) |  |  |
| EA | 8.5 | 8.38 | 0.042 | 0.838 | 8.5238±4.04992 | 8.4255±2.91267 | 0.026 | 0.873 |
| PA | 7.4419 | 6.88 | 0.858 | 0.356 | 7.8333±4.19591 | 6.9681±2.97824 | 1.883 | 0.172 |
| SA | 6.5349 | 6.28 | 0.258 | 0.612 | 6.8095±3.28513 | 6.2766±2.57533 | 1.043 | 0.309 |
| EN | 13.2442 | 12.04 | 1.942 | 0.166 | 12.5±4.98412 | 12.9362±4.84781 | 0.231 | 0.632 |
| PN | 10.814 | 10.88 | 0.029 | 0.865 | 10.8333±2.305 | 10.8404±2.12159 | 0 | 0.986 |

Abbreviations: CTQ, Childhood Trauma Questionnaire; FH, family history; EA, emotional abuse; PA, physical abuse; SA, sexual abuse; EN, emotional neglect; PN, physical neglect

**Supplementary Table 2. Analysis of MCCB and FH**

|  | Cognitive defect | Processing speed | Language learning and memory | Visual learning and memory | Social cognition | Attention and alertness | Working memory |
| --- | --- | --- | --- | --- | --- | --- | --- |
| FH+(200) | 9.27±2.170 | 134.05±26.634 | 43.76±10.539 | 43.60±11.719 | 47.48±11.986 | 41.79±10.969 | 89.95±20.904 |
| FH-(89) | 9.00±1.745 | 139.56±24.784 | 45.87±12.667 | 43.15±11.907 | 48.83±12.072 | 42.07±11.425 | 91.01±21.828 |
| F | 1.069 | 2.756 | 2.163 | 0.085 | 0.78 | 0.038 | 0.156 |
| *P* | 0.302 | 0.098 | 0.143 | 0.771 | 0.378 | 0.845 | 0.693 |

Abbreviations: MCCB, Matrics Consensus Cognitive Battery; FH+, with family history of psychosis; FH-, without family history of psychosis;

**Supplementary Table 3. The ANCOVA of PANSS and MCCB in terms of genotype**

|  | rs2303579 | rs3088077 | rs7162435 | rs11550869 | rs62043855 |
| --- | --- | --- | --- | --- | --- |
| Positive | F=3.707, ***P*=0.026*** | F=0.495, *P***=**0.610 | F=0.183, *P***=**0.833 | F=0.418, *P***=**0.659 | F=1.106, *P***=**0.332 |
| Negative | F=0.820, *P***=**0.442 | F=0.579, *P***=**0.561 | F=0.733, *P***=**0.481 | F=0.859, *P***=**0.425 | F=0.445, *P***=**0.641 |
| Excitement | F=1.375, *P***=**0.255 | F=3.305, ***P*=0.038*** | F=5.324, ***P*=0.005*** | F=1.592, *P***=**0.205 | F=0.656, *P***=**0.520 |
| Anxiety depression | F=1.260, *P***=**0.285 | F=0.516, *P***=**0.598 | F=0.503, *P***=**0.605 | F=0.529, *P***=**0.590 | F=0.713, *P***=**0.491 |
| Cognitive defect | F=1.721, *P***=**0.181 | F=0.661, *P***=**0.517 | F=2.811, *P***=**0.062 | F=0.593, *P***=**0.553 | F=0.522, *P***=**0.594 |
| Processing speed | F=2.837, *P***=**0.060 | F=111, *P***=**0.895 | F=0.173, *P***=**0.841 | F=0.487, *P***=**0.615 | F=2.649, *P***=**0.072 |
| Language learning and memory | F=1.388, *P***=**0.251 | F=0.503, *P***=**0.605 | F=0.280, *P***=**0.756 | F=0.127, *P***=**0.881 | F=1.046, *P***=**0.353 |
| Reasoning and problem solving skills | F=1.297, *P***=**0.275 | F=1.520, *P***=**0.221 | F=1.313, *P***=**0.271 | F=0.007, *P***=**0.993 | F=2.936, *P***=**0.055 |
| Visual learning and memory | F=0.502, *P***=**0.606 | F=0.380, *P***=**0.685 | F=0.873, *P***=**0.419 | F=0.031, *P***=**0.969 | F=1.690, *P***=**0.186 |
| Social cognition | F=0.171, *P***=**0.843 | F=0.636, *P***=**0.530 | F=0.338, *P***=**0.714 | F=0.198, *P***=**0.820 | F=0.009, *P***=**0.991 |
| Attention and alertness | F=1.031, *P***=**0.358 | F=0.517, *P***=**0.597 | F=0.187, *P***=**0.830 | F=0.148, *P***=**0.862 | F=0.519, *P***=**0.595 |
| Working memory | F=1.550, *P***=**0.214 | F=0.328, *P***=**0.721 | F=0.014, *P***=**0.986 | F=0.327, *P***=**0.721 | F=1.457, *P***=**0.235 |

^*^ *P*<0.05

Abbreviations: ANCOVA, analysis of covariance; PANSS,The Positive and Negative Syndrome Scale; MCCB, Matrics Consensus Cognitive Battery.

**Supplementary Table 4. Analysis of PANSS and** CTQ

| CTQ | | Positive | Negative | Excitement | Anxiety depression | Cognitive defect |
| --- | --- | --- | --- | --- | --- | --- |
| EA | None(69) | 32.17±4.376 | 31.16±7.907 | 13.59±4.041 | 12.86±3.405 | 8.87±1.940 |
|  | Exposed(67) | 33.19±4.800 | 32.88±7.776 | 14.73±4.561 | 12.27±3.231 | 9.01±2.164 |
| F |  | 1.679 | 1.637 | 2.371 | 1.06 | 0.17 |
| *P* |  | 0.197 | 0.203 | 0.126 | 0.305 | 0.68 |
| PA | None(99) | 32.44±4.529 | 31.55±7.679 | 13.73±3.966 | 12.68±3.377 | 8.87±2.024 |
|  | Exposed(37) | 33.30±4.795 | 33.24±8.311 | 15.30±5.055 | 12.27±3.194 | 9.14±2.123 |
| F |  | 0.925 | 1.259 | 3.614 | 0.402 | 0.455 |
| *P* |  | 0.338 | 0.264 | 0.059 | 0.527 | 0.501 |
| SA | None(81) | 32.77±4.643 | 31.22±7.866 | 13.80±3.916 | 12.62±3.444 | 8.69±1.934 |
|  | Exposed(55) | 32.55±4.578 | 33.16±7.781 | 14.67±4.861 | 12.49±3.162 | 9.31±2.168 |
| F |  | 0.074 | 2.013 | 1.328 | 0.047 | 3.029 |
| *P* |  | 0.786 | 0.158 | 0.251 | 0.829 | 0.084 |
| EN | None(36) | 32.75±5.073 | 31.83±7.311 | 13.39±3.643 | 13.11±3.353 | 9.08±2.048 |
|  | Exposed(100) | 32.65±4.446 | 32.07±8.084 | 14.43±4.533 | 12.37±3.305 | 8.89±2.054 |
| F |  | 0.012 | 0.024 | 1.538 | 1.321 | 0.235 |
| *P* |  | 0.911 | 0.878 | 0.217 | 0.252 | 0.629 |
| PN | None(7) | 33.00±4.203 | 33.00±8.287 | 14.57±4.541 | 12.57±2.637 | 9.71±1.380 |
|  | Exposed(129) | 32.66±4.636 | 31.95±7.868 | 14.13±4.333 | 12.57±3.363 | 8.90±2.072 |
| F |  | 0.036 | 0.117 | 0.068 | 0.000018 | 1.053 |
| *P* |  | 0.849 | 0.733 | 0.795 | 0.997 | 0.307 |

Abbreviations: PANSS,The Positive and Negative Syndrome Scale; CTQ, Childhood Trauma Questionnaire; FH, family history; EA, emotional abuse; PA, physical abuse; SA, sexual abuse; EN, emotional neglect; PN, physical neglect
